# A study on the correlation between hemoglobin concentration and the storage quality of suspended red blood cells prepared from the whole blood of Tibetan male residents

**DOI:** 10.3389/fmed.2022.1062778

**Published:** 2023-01-20

**Authors:** Rui Zhong, Xiaodong Wu, Zhijuan Liu, Zeng He, Xuejun Zhang, Jiaxin Liu, Ye Cao, Hong Wang

**Affiliations:** ^1^Peking Union Medical College, Institute of Blood Transfusion, Chinese Academy of Medical Science, Chengdu, Sichuan, China; ^2^Department of Critical Care Medicine, Hospital of Chengdu Office of People's Government of Tibetan Autonomous Region, Chengdu, China; ^3^Department of Clinical Laboratory, People's Hospital of Tibet Autonomous Region, Lhasa, Tibet, China; ^4^Department of Biobank, Hospital of Chengdu Office of People's Government of Tibetan Autonomous Region, Chengdu, China

**Keywords:** high altitude, hemoglobin concentration, storage quality, suspended red blood cells, erythrocyte metabolism

## Abstract

**Background:**

Previous studies reported that the blood of Tibetans living at different altitudes may vary slightly. There is evidence that the harsh environmental conditions at high altitudes, such as low pressure and hypoxia, may affect the morphology and hemorheology of red blood cells (RBCs). Hypoxia would increase the levels of hemoglobin ([Hb]) and hematocrit (Hct), potentially increasing blood hyperviscosity and compromising blood collection and transfusions. Therefore, it is critical to investigate the *in vitro* storage quality of Tibetan RBCs.

**Objectives:**

In this study, the *in vitro* quality of suspended RBCs (SRBCs) prepared from whole blood (WB) of Tibetan residents with varying Hb concentrations ([Hb]) was measured during storage, and the relationship between the major factors in RBC storage and [Hb] was studied.

**Materials and methods:**

The WB of Tibetan men was divided into three groups based on [Hb] levels (group A: 120 < Hb ≤ 185 g/L; group B: 185 < Hb ≤ 210 g/L; group C: Hb > 210 g/L). The SRBCs prepared from WB were examined aseptically on days 1, 14, 21, and 35 after storage.

**Results:**

[Hb] was not correlated with mean corpuscular volume (MCV), adenosine triphosphate (ATP), pH, P50, and hemolysis. There was a moderate or strong negative association between platelets (PLT) and [Hb] from days 1 to 35, and the PLT number of group C was lower than group A during storage. Group C had the highest change rates of electrolytes, glucose, and lactate, and there were moderate or strong positive correlations between lactate and [Hb] (*r* = 0.3772, *p* = 0.0045), glucose and [Hb] (*r* = 0.5845, *p* < 0.0001), Na^+^ and [Hb] (*r* = 0.3966, *p* = 0.0027), and K^+^ and [Hb] (*r* = 0.4885, *p* = 0.0002). Group B had the highest change rates of 2,3-DPG on day 35, and there was a negative correlation between 2,3-DPG and [Hb] (*r* = −0.4933, *p* = 0.0001).

**Conclusions:**

These new data on the [Hb] could have implications for researchers wishing to study the storage quality of Tibetan SRBCs, particularly in the context of erythrocyte metabolism, and we propose finding a new, suitable alternative solution for plateau SRBCs, particularly the blood with [Hb] greater than 185 g/L. Our results could have important implications for researchers wishing to study the potential framework of high-altitude-induced SRBC storage lesions.

## Introduction

More than 140 million people worldwide live at an altitude of more than 2,500 m for extended periods of time, but environmental conditions at high altitudes, such as low temperature, low pressure, and hypoxia, may affect the morphology and phenotype of blood cells (1–3). Due to the lack of oxygen in high-altitude areas, the bone marrow was stimulated by erythropoietin, which increases the production of red blood cells (RBCs) (4, 5). Hemoglobin concentration ([Hb]) was found to increase with the number of RBCs, with an average value of 17–20 g/100 ml, compared with an average value of 12–16 g/100 ml at the sea level (6–8). However, the [Hb] values reported for people living at different altitudes were not in the same range. The Qinghai Tibet Plateau, located in the northwest of China, is the world's largest and highest plateau with an average altitude of more than 4,000 m (9). The researchers found that the [Hb] of most Tibetan men ranged from 15 to 16 g/100 ml, with the [Hb] increasing with an increase in altitude (9). As another example, due to long-term residence in Tibet at extremely high altitudes (>4,000 m), the [Hb] of some Tibetans was higher than the main diagnostic criteria for polycythemia vera (PV) (men > 185 g/L, women > 165 g/L) or even higher than that for high-altitude polycythemia (HAPC) (men > 210 g/L, women > 190 g/L) (10). Thus, excessive erythropoiesis, high hematocrit (Hct), and an excessive amount of [Hb] in the blood of Tibetans would cause blood hyperviscosity, compromising blood collection and transfusions (11–13).

Suspended RBCs (SRBCs) are the most commonly used transfusion blood products prepared from donated whole blood (WB) (14, 15). Our previous study (16) showed that the Hct level of SRBCs prepared from the WB of Tibetan migrants exceeded 60% and that the storage quality of SRBCs prepared by high-altitude migrants was inferior to that of SRBCs prepared from lowlands. For example, the metabolic rate of SRBCs (including electrolytes and lactic acid) was higher at high altitudes than at low altitudes. These findings were consistent with the osmotic fragility and hemolysis of plateau SRBCs during the whole storage period. These changes might be related to higher [Hb] of WB prepared from the WB of Tibetan migrants. However, because Tibetans might have a wide range of [Hb], the biological changes and clinical blood transfusion compatibility of Tibetan SRBCs stored for 35 days had not been evaluated. Therefore, the storage quality of SRBCs prepared from WB of Tibetan residents must be investigated.

In this study, the *in vitro* quality of SRBCs prepared from Tibetan residents with varying [Hb] during storage was determined. It was reported (9) that the [Hb] of men was higher than that of women in the Tibetan adult population and increased with elevation. Gender plays an important role in the influence of altitude on [Hb]. However, the number of women with [Hb] >165 g/L was much lower than the number of men with [Hb] >185 g/L, and it was difficult to draw blood in the same proportion for men and women. Thus, the storage quality of SRBCs prepared from the blood of Tibetan men was initially studied. The [Hb] of WB from Tibetan men was divided into three groups; the [Hb] in group A was 120–185 g/L, the [Hb] in group B was 186–210 g/L, and the [Hb] in group C was >210 g/L. A number of assays for pH, percent hemolysis, electrolytes and glucose in the supernatant, and lactate production were tested, while more detailed assessments of RBC metabolism and quality, including adenosine triphosphate (ATP), 2,3-DPG, and oxygen affinity (P50), were also used. Furthermore, the purpose of this study was to investigate the effects of high [Hb] on the storage quality of SRBCs from the Tibetan male population. Our findings provide insights into the mechanism of high-altitude-induced SRBC storage lesions.

## Materials and methods

### WB collection and RBC processing and storage

Whole blood was collected according to standard procedures, and the volunteers were from the Hospital of Chengdu Office of the People's Government of Tibetan Autonomous Region and People's Hospital of Tibet Autonomous Region between December 2018 and August 2019. All 55 Tibetan male volunteers aged between 19 and 55 years with no organic disease had lived at high altitudes (>3,000 m) for more than a decade. The inclusion criteria were as follows: all volunteers were ≥18 years and unrelated. Patients with a history of thrombosis or bleeding, the use of oral anticoagulants, liver disease, HIV infection, pregnancy, diabetes, and others were excluded from this study. The study protocol was approved by the Institute of Blood Transfusion Ethics Committee (Registration number: 201712), and the authors had access to the information that could identify individual participants during or after data collection. The samples were divided into three groups with varying [Hb] of WB from Tibetan men, and basic information on volunteers is presented in Supplementary Table S1.

Whole blood (200 ml ± 10%) was collected into a blood collection kit containing a citric acid-phosphate dextrose-adenine 1 (CPDA-1) anticoagulant (28 ml) (Sichuan Nigale Biomedical Company, China). The formulation of anticoagulant CPDA-1 is listed in Supplementary Table S2. Within 1 h after collection, each WB bag was centrifuged at 3,500 × g for 10 min to remove the plasma, and 50 ml of mannitol-adenine-phosphate (MAP) was added (see Supplementary Table S2 for the composition of the additive solution) to the remaining RBCs to obtain SRBCs. SRBCs were stored at 2–6°C under standard blood bank conditions (17) and analyzed on days 1, 14, 21, and 35 of storage.

### Routine assessment of RBC quality

Samples were collected on days 1, 14, 21, and 35 of storage, blood SRBCs were examined using an automatic blood analyzer (XT-1800i; Mindray, Shenzhen, China), and the levels of RBCs, hemoglobin, Hct, platelets (PLT), and mean corpuscular volume (MCV) were measured. The supernatant was obtained by centrifuging SRBCs at 3,500 × g for 10 min at 4°C, and potassium (K^+^), sodium (Na^+^), glucose, and lactate concentration in the supernatant were determined using an automatic biochemical analyzer (7180; Hitachi Co., Ltd., Japan). At room temperature (25°C ± 2°C), the extracellular pH was measured using a pH meter (FE20; Mettler Toledo, Switzerland). According to the recommended protocol (Roche Diagnostics, Mannheim, Germany), ATP and 2,3-DPG were evaluated using commercial kits.

### Hemolysis

In short, the absorbance of the supernatant at 505 nm was measured using a spectrophotometer (Ultrospec 6300 Pro; Amersham Biosciences, USA) to calculate the free Hb content. The hemolysis rate was expressed as the percentage of free hemoglobin content in total Hb after Hct correction.

### Oxygen affinity

When the blood oxygen saturation (P50) reached 50%, it was detected by a blood oxygen analyzer (TCS Scientific, USA). To obtain the suspension, 50 μl of RBC was added to the diluent. Oxygen was kept in suspension once the oxygen partial pressure (PO_2_) exceeded 13.33 kPa (100 mmHg). Oxygen was then turned off, and nitrogen was added until PO_2_ decreased by more than 0.67 kPa (5 mmHg). The oxygen production dissociation curve (ODC) was drawn after changes in PO_2_ and oxyhemoglobin were detected during the process. Finally, P50 was measured using ODC.

### Statistical analysis

The results were shown as mean ± standard deviation (SD) of repeated samples. Unless otherwise specified, each parameter must be tested at least three times. Analysis of variance (ANOVA) of repeated measures and the Bonferroni test were used to compare the three groups of SRBCs stored at the same time, and the impact of storage time on SRBCs was evaluated. Pearson's correlation analysis was used to check the correlation between storage parameters and [Hb]. A correlation coefficient (*r*) of 0.10–0.29 indicated a weak correlation, 0.30–0.49 indicated a moderate correlation, and 0.50–1.0 indicated a strong correlation. SPSS statistical software version 17.0 (SPSS, Inc., Chicago, Illinois, USA) was used for statistical analysis. A *p*-value of < 0.05 was considered statistically significant when using the 95% confidence interval (CI) [2.5–97.5%].

## Results

### Group C had high [Hb], Hct levels, and a low PLT number

**Figure 1:**
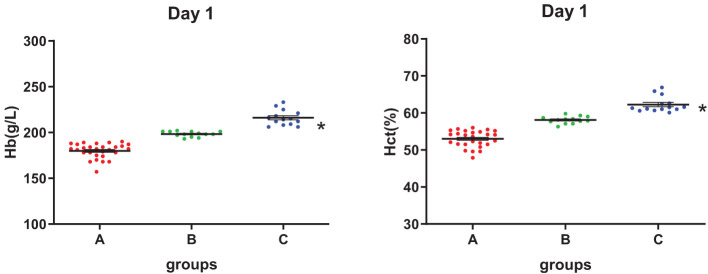
Hemoglobin concentration ([Hb]) and hematocrit (Hct) level of three groups on day 1. *Analysis of variance (ANOVA), *p* < 0.05 (*p* = 2.13E-9, 2.95E-20) for group C vs. group A.

As shown in Figure 1, the [Hb] of SRBCs in three groups was mainly distributed in the range of 180–210 g/L, and Hct was mainly located in the range of 50–60%, with [Hb] and Hct in group C being significantly higher than those in group A. All three groups had no significant difference in the MCV values at each storage test point (see Supplementary Table S3). The PLT number in group C was significantly lower than that in group A during storage, and the PLT number in all groups decreased with storage time (see Supplementary Table S3).

### The effect of [Hb] on MCV and PLT

**Figure 2:**
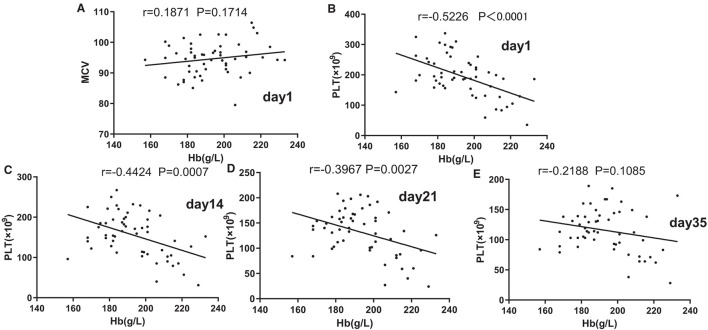
Associations of mean corpuscular volume (MCV) **(A)** and Hb and of platelets (PLT) and Hb on days 1 **(B)**, 14 **(C)**, 21, and **(D)** 35. **(E)** Pearson's partial correlation analysis was used.

Pearson's partial correlation analysis was used to investigate the effect of [Hb] on MCV and PLT, as shown in Figures 2A–E. No correlation was found between MCV and [Hb] on day 1 (*p* > 0.1). In contrast to MCV, from days 1 to 21, there was a strong or moderate negative association between PLT and [Hb] (*p* < 0.05).

### Group C had higher change rates of electrolytes, glucose, and lactate

**Figure 3:**
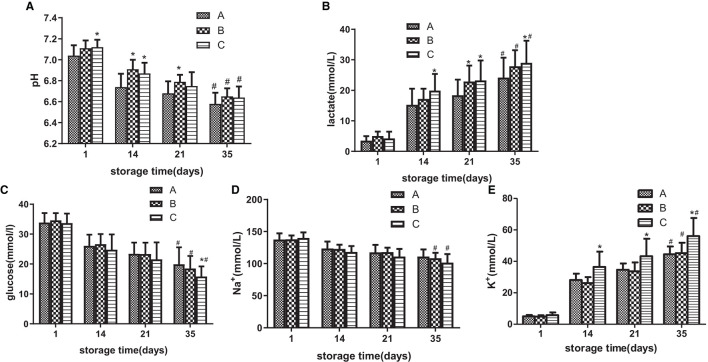
During the storage period of 35 days, the standard quality parameters of suspended red blood cells (SRBCs) stored in different groups were measured. pH **(A)**, lactic acid concentration **(B)**, glucose concentration **(C)** Na^+^ concentration in the supernatant **(D)**, and K^+^ level in the supernatant **(E)**. *ANOVA *p* < 0.05 [**(A)** *p* = 0.047, 4.91E-5, 0.004, 0.010, **(B)** *p* = 0.004, 0.014, 0.006, 0.012, **(C)** *p* = 0.009, **(E)** *p* = 9.83E-10, 0.001, 9.72E-5] in comparison to group A on days 1, 14, 21, and 35, respectively. # ANOVA *p* < 0.05 [**(A)** *p* = 9.83E-10, 8.22E-10, 0.001, **(B)** *p* = 9.95 E-12, 8.87 E-12, 9.62 E-12, **(C)** *p* = 0.010, 0.006, 0.003, **(D)** *p* = 0.035, 0.026, **(E)** *p* = 8.84E-10, 9.21E-10, 9.43E-10] for all parameters on day 1 vs. day 35.

On day 1, pH in groups A, B, and C was 7.04, 7.11, and 7.12, respectively, and on day 35, it decreased to 6.57, 6.65, and 6.64. From days 1 to 14, pH in group C was higher than that in group A (*p* < 0.05), from days 14 to 21, pH in group B was higher than that in group A, and on day 35, there was no significant difference among all three groups (Figure 3A). From days 14 to 35, lactate production in group C was higher than that in group A (*p* < 0.05; Figure 3B). The average blood glucose level in all three groups decreased with a prolonged storage time. Except for day 35, there were no significant differences among the three groups, and the blood glucose level in group C was lower than that in group A (Figure 3C). From days 14 to 35, group C had a higher K^+^ level in the supernatant than in group A. During the whole storage process, there was no significant difference in the Na^+^ level among the three groups (Figures 3D, E).

The red blood cell count was significantly higher in group C than in group A, and erythrocyte metabolic parameters such as glucose, lactic acid, Na^+^, and K^+^ were related to the number of RBCs. To exclude the influence of the number of RBCs on these parameters, the metabolic rate of RBCs was calculated using the previously reported formula (16). The following formula was used to calculate the RBC metabolic rate:


$$\begin{array}{l} {\text{R}}_{\text{x}}=\frac{{\text{V}}_{\text{x}1}-{\text{V}}_{\text{x}35}}{{\text{N}}_{\text{rbc}}}, \end{array}$$

where *R* denotes the rate of change of these parameters during the whole storage period, *x* denotes the parameters, including glucose, lactic acid, Na^+^, and K^+^, *V*_x1_ denotes the parameter value on day 1 of storage, *V*_x35_ denotes the parameter value on day 35 of storage, and *N*_RBC_ denotes the number of RBCs on day 1 of storage.

The results (Table 1) revealed that group C had higher change rates of all these parameters than group A and that group B had higher change rates of glucose and lactate than group A.

**Table 1:** The change rate of Na^+^, K^+^, glucose, and lactate in suspended red blood cells (SRBCs) during the whole storage period.

**SRBC units**	**Na^+^**	**K^+^**	**Glucose**	**Lactate**
	**(mmol/L/rbc** × **10**^12^**)**	**(mmol/L/rbc** × **10**^12^**)**	**(mmol/L/rbc** × **10**^12^**)**	**(mmol/L/rbc** × **10**^12^**)**
A	4.85 ± 0.58	6.89 ± 0.52	2.44 ± 0.73	3.53 ± 0.71
B	4.96 ± 1.03	6.96 ± 0.85	2.78 ± 0.68^*^	3.91 ± 0.90*
C	5.90 ± 1.24^*^	7.91 ± 1.04^*^	2.91 ± 0.59^*^	3.91 ± 0.82^*^

Data were reported as mean ± standard deviation (SD). ^*^Analysis of variance (ANOVA) *p* < 0.05 (Na^+^: *p* = 0.38, K^+^: *p* = 0.041, glucose: *p* = 0.042, 0.038, lactate: *p* = 0.045, 0.043) in comparison to group A.

### Effect of [Hb] on change amounts of pH, lactate, glucose, Na^+^, and K^+^ during storage

**Figure 4:**
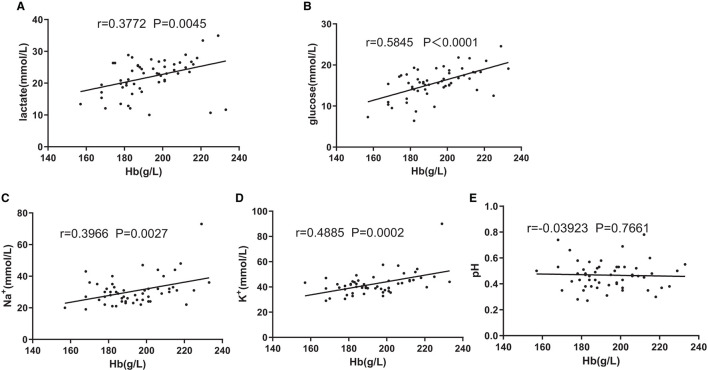
Associations of change amount during the storage of lactate **(A)** and [Hb], glucose **(B)** and [Hb], Na^+^ **(C)** and [Hb], K^+^ **(D)** and [Hb], and pH and [Hb] **(E)**. Pearson's partial correlation analysis was used.

As shown in Figures 3B–E and Table 1, group C significantly differed from group A in terms of lactate, glucose, Na^+^, and K^+^ concentrations at the end of storage and in the change rate during storage. Then, partial correlation analysis was used to investigate the relationship between the metabolic amount of RBC (the value on day 35 minus the value on day 1 or the value on day 1 minus the value on day 35) and [Hb] and the positive correlations between lactate and [Hb] (*r* = 0.3772, *p* < 0.01, Figure 4A), between glucose and [Hb] (*r* = 0.5845, *p* < 0.0001, Figure 4B), between Na^+^ and [Hb] (*r* = 0.3966, *p* < 0.01, Figure 4C), and between K^+^ and [Hb] (*r* = 0.4885, *p* < 0.001, Figure 4D) were found. In contrast, although pH was higher in groups B and C than that in group A from days 1 to 21, there was no correlation between pH change value and [Hb] (*r* = 0.03923, *p* > 0.1, Figure 4E).

### Group B had higher 2,3-DPG on day 35, and there was no significant difference in ATP and P50 among the three groups

**Figure 5:**
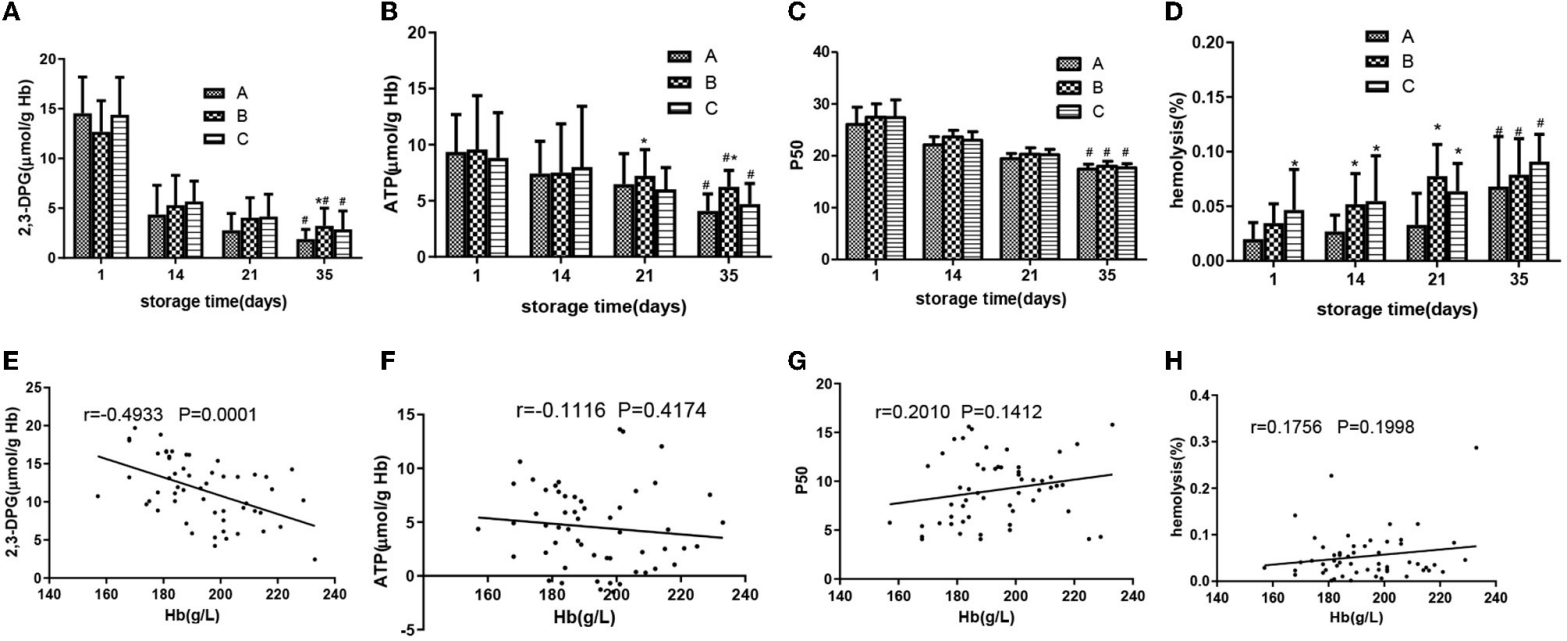
SRBCs in different groups were measured during a 35-day storage period. 2,3-DPG **(A)**, adenosine triphosphate (ATP) **(B)**, P50 **(C)**, and hemolysis **(D)**. Associations of change amount during the storage of 2,3-DPG **(E)** and Hb, ATP **(F)** and Hb, P50 **(G)**, hemolysis **(H)**, and Hb. Pearson's partial correlation analysis was used. *ANOVA *p* < 0.05 [**(A)** *p* = 0.030, **(B)** *p* = 0.013, 0.041, 0.019, 8.84E-10, **(D)** *p* = 0.004, 0.034, 0.011, 9.76E-5, 0.009] in comparison to group A on days 1, 14, 21, and 35. # ANOVA *p* < 0.05 [**(A)** *p* = 5.45E-6, 0.010, 4.78E-6, **(B)** *p* = 0.020, 0.046, 0.035, **(C)** *p* = 0.037, 0.032, 0.035, **(D)** *p* = 0.26, 0.031, 0.028] for all parameters on day 1 vs. day 35.

On day 35, group B had a higher level of 2.3-DPG than group A (*p* < 0.05) (Figure 5A). Except for group B, which had higher ATP levels than group A on days 21 and 35, there was no significant difference in the ATP levels between groups C and A (Figure 5B). As shown in Figure 5C, oxygen affinity increased rapidly during storage, and P50 (partial pressure of oxygen when hemoglobin was half saturated) decreased. During the whole storage period, there was no significant difference in P50 values among the three groups. Although the allowable range of hemolysis was 0.8 and 1.0% in Europe and North America, respectively (17) (Figure 5D), during the storage of all RBCs, group C had a higher hemolytic value than group A on days 1, 14, and 21 (*p* < 0.05), group B had a higher hemolysis value than group A on days 14 and 35, and there were no significant differences among the three groups on day 35.

### Effect of [Hb] on the amount of changes in 2,3-DPG, ATP, P50, and hemolysis during storage

Partial correlation analysis was used to investigate the relationship between the change amount of 2,3-DPG, ATP, P50, and hemolysis (the value on day 1 minus the value on day 35) and [Hb], and a negative correlation between 2,3-DPG and [Hb] (*r* = −0.4933, *p* < 0.001, Figure 5E) was found. In contrast, there were no correlations between ATP change value and [Hb] (*r* = 0.1116, *p* >0.05, Figure 5F) and P50 change value and [Hb] (*r* = 0.2010, *p* > 0.1, Figure 5G). There was no correlation between hemolysis change value and [Hb] (*r* = 1,756, *p* > 0.1, Figure 5H).

## Discussion

It was reported that hypoxia caused hemoglobin (Hb) and Hct levels to increase significantly and stabilize at relatively high levels after individuals ascended to high altitudes (18). Our previous study found that altitude might play a major role in changes in RBC storage, and the storage quality of SRBCs from Tibetan Plateau migrants was significantly different from the residents in the Deyang lowland (16). However, the relationship between the storage quality of SRBCs prepared by WB from Tibetan natives with different [Hb] was still unclear. In this study, the *in vitro* quality of SRBCs prepared from Tibetan residents with different [Hb] during storage was measured, and the results showed that MCV, ATP, P50, and the pH value were not associated with [Hb], while major factors, such as PLT, glucose, lactate, Na^+^, K^+^, and 2,3-DPG, played a critical role in RBC storage lesions and were found to be related to [Hb] in the Tibetan population.

Because RBCs were key participants in systemic oxygen transport, they played a visible role in hypoxia adaptation (19). RBCs would undergo a variety of metabolic, structural, and morphological changes during storage (20). MCV is a measure of the overall change in RBC volume. Our data suggested that there was no difference in MCV among the three groups during the whole storage period and that there was no correlation with the storage time of each group. As expected, the results of our study showed that [Hb] was not associated with MCV (*r* = 0.0585, *p* > 0.1), indicating that [Hb] had no effect on the overall volume of RBCs.

Adenosine triphosphate, as an energy source, is crucial to the global function of the cells. ATP loss was related to firmer cell membranes, loss of vasodilatation function, exposure to phosphatidylserine (PS) on the outer lobe of the erythrocyte membrane, and decreased erythrocyte activity. The ATP levels should be >2.7 μmol/g Hb to have a 90% RBC survival rate of 90% (the *in vivo* 24-h recovery rate was 75% or higher) (21). ATP concentrations in the three groups gradually decreased during storage and exceeded 2.7 μmol/g Hb at the end of storage, and ATP reduction during storage was not associated with [Hb].

Under normal conditions, the P50 value of human RBC was 26–27 mmHg. It was found that, at high altitudes, P50 increased and the affinity of hemoglobin for oxygen decreased, implying that oxygen bound by hemoglobin was more effective for body tissues (22, 23). In this study, P50 was found to be in the normal range in the three groups of Tibetan permanent residents and decreased with storage time, resulting in a decrease in oxygen release from RBCs to tissues. In addition, there was no significant difference among the three groups during the whole storage time, and there was no correlation between P50 and [Hb].

Platelets are the indispensable key factors in the process of thrombosis. Our study showed that PLT counts among the three groups decreased as storage time increased. This result might be related to storage temperature and time. When the storage temperature of PLT was lower than 18°C, the lipid bilayer membrane would undergo a phase transition, resulting in the aggregation of surface glycoproteins (24). PLT were lost because they were unable to find receptors within their limited shelf-life. In addition, PLT counts had a negative correlation with [Hb] in HA volunteers during storage time, and group C had lower PLT counts. The results were different from those of Zhang et al. (25), who found no correlations between the PLT count and [Hb] in HA volunteers. Different results might be obtained due to the different [Hb] groups. Other reports showed that the PLT count in HA was lower than that in low-altitude areas (26–28). However, other researchers demonstrated an increase in PLT counts in HA (29–31). These results could be explained by the fact that PLT count was related to [Hb]. High [Hb] directly causes high blood viscosity and slow blood flow, which may result in the activation and consumption of a large number of PLT and a reduction in the number of PLT in circulating blood.

When the glucose concentration decreased during storage, the content of lactic acid and pyruvic acid increased, leading to a decrease in pH and the consumption of ATP and 2,3-DPG in RBCs. Our results showed that the amount of glucose depletion and lactate accumulation was positively related to [Hb], with higher [Hb] inducing a faster rate of glucose depletion and lactate accumulation. This could be due to the tighter accumulation of RBCs, leading to faster glucose consumption and harder recovery *in vivo* (32, 33). However, more lactate accumulation in group C did not result in more decrease in pH, and the amount of pH decrease was not associated with [Hb]. Group C had a higher pH value than group A on days 1, 14, and 21, and there was no significant difference at the end of storage. Kurup et al. (34) found that pH might be related to the level of 2,3-DPG during the storage of RBCs. pH > 7.0 was conducive to the synthesis of 2,3-DPG, and pH < 7.0 was conducive to its degradation. Meanwhile, other studies indicated that the balance of ATP and 2,3-DPG synthesis was pH dependent, where a pH value of < 7.2 was conducive to the decomposition of 2,3-DPG and CO_2_ levels (35). Although there was no initial significant difference in 2,3-DPG among the three groups, our results were partly in agreement with these reports because, at the end of storage, 2,3-DPG in groups B and C was higher than that in group A, and the amount of 2,3-DPG reduction during storage was inversely correlated with [Hb]. The higher 2,3-DPG in group C might be related to a higher initial pH value. Because 2,3-DPG regulated oxygen transport to tissues, it became crucial for oxygen transport after a blood transfusion.

The intracellular K^+^ level plays a major role in maintaining cell water content. The loss of K^+^ in RBCs leads to dehydration, resulting in increased density, shape changes, and rheological defects (36). RBCs stored in group C had higher extracellular K^+^ levels and lower Na^+^ levels compared with RBCs stored in group A at the end of storage, and extracellular K^+^ production and Na^+^ consumption during storage were positively related to [Hb]. Hypoxia could cause K^+^ outflow and Ca^2+^ inflow as well as an increase in ATP decomposition and a decrease in ATP synthesis, resulting in an energy deficit. When the ATP content decreased, the Na/K membrane pumps stopped working or a small number of RBCs had burst during storage, inducing intracellular K to leak into the storage media. Refrigeration conditions paralyzed the ion pump and reduced its activity, resulting in the leakage of K^+^ (37). Similarly, the erythrocyte membrane structure was destroyed and deformability was reduced after the activation of protease and phospholipase. However, even after excluding the influence of RBCs, the change amount of K^+^ and Na^+^ were still higher in group C than in group A, which indicated that an excessive increase in [Hb] and RBCs modified some components of the RBC membrane, increased the surface area and abnormal morphology of RBC, alternating the rigidity of RBCs and abnormalities in membrane contractile proteins.

Mannitol-adenine-phosphate is an RBC preservation solution widely used in most Asian countries, including China. The main ingredients included sodium citrate, glucose, adenine, mannitol, and others. In the conventional use scheme, 100 ml of MAP was added to RBCs centrifuged from 400 ml of WB to prepare SRBCs, with a preservation period of 35 days. In our study, the Hct values of SRBCs in groups B and C were more than 55%, indicating high blood viscosity and thus a high risk of clinical transfusion. In our previous study, the volume of MAP was increased to prepare plateau SRBCs, and the results indicated that the number of RBCs, [Hb], Hct, and blood viscosity were all significantly reduced, facilitating clinical transfusion. In addition, increased AS volume could delay glucose consumption and RBC hemolysis. As a result, the preservation quality of SRBC at high altitudes was further improved. Therefore, we proposed that the volume of MAP was increased to prepare plateau SRBCs or that the storage solution formula needed to be adjusted. For example, adding ascorbic acid could preserve the membrane integrity of RBCs. New alternatives to plateau SRBCs should be explored, especially for people with [Hb] levels >185 g/L.

## Conclusion

In conclusion, this study began to evaluate the effect of Hb concentration on the main RBC parameters of SRBCs prepared from WB of Tibetan men during storage. These results indicated that [Hb] might not be correlated with MCV, pH, ATP, P50, and hemolysis; PLT, 2,3-DPG, and [Hb] had a negative association; lactate, glucose, and [Hb] had a high positive correlation; and Na^+^, K^+^, and [Hb] had a moderate positive correlation. These new data on the [Hb] might have implications for researchers wishing to study the storage quality of RBCs, particularly in the context of erythrocyte metabolism, and our results might have important implications for researchers wishing to study the potential framework of high-altitude-induced SRBC storage lesions. In addition, we suggest finding a new, suitable alternative solution for plateau SRBCs, especially for people with [Hb] >185 g/L. Our findings highlight the importance of continuously exploring novel frameworks to improve the storage of SRBCs at high altitudes.

## Data availability statement

The raw data supporting the conclusions of this article will be made available by the authors, without undue reservation.

## Ethics statement

The studies involving human participants were reviewed and approved by Evaluation Report of Ethics Committee of Blood Transfusion Institute, Chinese Academy of Medical Sciences (No: 201712) Institute of Blood Transfusion, Chinese Academy of Medical Sciences. The patients/participants provided their written informed consent to participate in this study.

## Author contributions

RZ contributed to data interpretation and drafting of the paper. HW, JL, and XW contributed to the study conceptualization and design. YC, ZH, ZL, and XZ participated in the acquisition of the data and critical revision of the draft. All authors contributed to the article and approved the submitted version.
