# Supplementary material for: A study on the correlation between hemoglobin concentration and the storage quality of suspended red blood cells prepared from the whole blood of Tibetan male residents
Source: Front Med (Lausanne). 2023 Jan 20;9:1062778. doi: 10.3389/fmed.2022.1062778 (PMC9895097; doi:10.3389/fmed.2022.1062778)
Supplement: Supplementary file 1 [file Table_1.DOC]

**SI Table 1. The sample grouping information of WB in this study**

| Group | n | Hb （g/L) | Age(yrs) | Altitude(meters) |
| --- | --- | --- | --- | --- |
| A | 28 | 120-185 | 39±10 | 3733±380 |
| B | 13 | 186-210 | 40±9 | 3700±295 |
| C | 14 | >210 | 44±7 | 3686±281 |

**SI** Table 2. The composition of CPDA-1 and MAP

| composition | CPDA-1 | MAP |
| --- | --- | --- |
| sodium citrate (mM) | 89 | 5.1 |
| acid citrate (mM) | 17 | 1.0 |
| sodium phosphate (mM) | 5.8 | _ |
| NaH_2_PO_4_ (mM) | _ | 7.8 |
| Glucose (mM) | 177 | 40 |
| NaCl (mM) | _ | 85 |
| Adenine (mM) | 2.0 | 1.0 |
| Mannitol (mM) | _ | 80 |

**SI Table 3. MCV and PLT number for 3 groups throughout the entire storage period**

| Storage time (days) | MCV (FL) | | |  | PLT |  |
| --- | --- | --- | --- | --- | --- | --- |
|  | A | B | C | A | B | C |
| 1 | 92.81±4.91 | 94.89±3.90 | 95.69±6.69 | 227.48±60.13 | 191.21±38.99^*^ | 123.71±54.31^*^ |
| 14 | 92.43±4.74 | 94.98±5.09 | 95.77±6.28 | 182.38±63.87 | 158.26±44.22 | 99.71±46.66^*^ |
| 21 | 92.32±5.35 | 93.73±5.08 | 95.69±4.29 | 152.64±48.94 | 143.58±38.73 | 83.47±40.77^*^ |
| 35 | 93.18±4.69 | 94.24±4.03 | 95.05±5.03 | 120.90±36.91^#^ | 127.83±34.92^#^ | 81.94±42.95^*#^ |

* Significant results (P＜0.05) in comparison to A group on day 1,14,21 and 35. # ANOVA p < 0.05 for groups on day 1 vs day 35.
